# The Influence of Maternal Impressions on the Fœtus

**Published:** 1889-01

**Authors:** Frederick L. Classen

**Affiliations:** Albany, N. Y.


ARTICLE III.

### THE INFLUENCE OF MATERNAL IMPRESSIONS ON THE FŒTUS.

BY FREDERICK L. CLASSEN, M. D., ALBANY, N. Y.

[Read before the Medical Society of the County of Albany, Wednesday Evening, November 14, 1888.]

The belief, held by many, as far back as Plato, is becoming stronger now, that the condition of the mother's mind has a great influence upon her unborn child; that not only deformities may result from maternal impressions, but that modifications of the moral and intellectual character of the child, as manifested in after years, may have their origin in the mental state of the pregnant woman. Hence should be guarded from all injurious influences and painful sights. There are some women so highly impressionable as to react to the slightest nerve stimulus; and from the moment of fecundation the ovum is exposed to various influences which may alter its normal development.

During my first year of practice, a lady, advanced to the eighth month of pregnancy, called at my office, wishing to have a tooth extracted. She asked me if it would do any harm to her unborn child. I told her I did not think it would. I delivered her one month later of a dead child. I have always thought that the death of the child was caused by the extraction of the tooth.

Having this case in mind, I have always deemed it inadvisable to do any tooth-pulling for a pregnant woman. And yet, about three months ago, a lady, of an extremely nervous temperament, wished to have six decayed teeth extracted. She asked me if it would injure her in any way, as she had then gone about three weeks over her menstrual period. I told her it might produce an abortion. "Well," said she, "I will have to run the risk, as I cannot stand the pain." She was put under ether, and the teeth extracted. No abortion followed. She now asks whether the child will be marked.

Many writers are opposed to the theory of maternal impressions, and claim that the deformity is due to an arrest of development or to coincidence. I would mention Dr. J. G. Fisher, of Sing Sing, N. Y., who prepared a very able and scientific paper entitled "Does Maternal Influence Have Constructive or Destructive Power in the Production of Malformations or Monstrosities at any Stage of Embryonic Development?" * I will quote some of his deductions:

1. "That traditional superstition has perpetuated the notion that malformations are the result of mental emotion."

2. "That the medical profession is in no inconsiderable degree responsible for the existence and continuance of this popular error."

3. "That various intense emotions are common with gestating women, and apprehensions of malformation of their offspring exists in the minds of a large portion, yet abnormal births are extremely rare."

4. "That there is nothing like law in the alleged results of maternal mental influence in the production of malformation."

5. "That the occasional apparent relation of cause and effect is due, in most instances, to accidents, which would be far less frequent if the facts could be obtained

---

* American Journal of Insanity, January, 1870.

*previously* instead of *subsequently* to the birth of the child."

6. "Such evidences are not sufficiently numerous and authentic to warrant a rational belief in the origin of monstrosities from the perturbed emotions of the mother's mind."

7. "Like causes produce like results, whereas we find that in a series of cases of any special variety of malformation mental emotions arising from a considerable number of dissimilar objects, even of the most diverse character, are assigned as the cause."

8. "In a large proportion of the cases of malformation no mental or even physical explanation is offered by the parents or friends."

9. "There is no relation between the number and character of these mental emotions and apprehensions of pregnant women and the actual frequency and variety of malformations."

10. "That some of the assumed causes are alleged to have operated upon the embryo or fœtus subsequently to the named period for the evolution of the part which is found to be the seat of the malformation, thereby implying a distinctive as well as a metamorphosing power."

12. "Malformations identical in kind and in degree recur again and again in the human subject, and admit of a systemic classification, definite and distinctive."

13. "Every form of malformation and monstrosity in the human subject, has had its exact morphological counterpart in the lower animals."

15. "The only rational and scientific explanation is to be found in pathological histology."

16. "Monstrosities are not the result of embryological and physiological laws; they are the products of embarrassments to normal development."

17. "Vices of conformation and monstrosities are due to either retarded or excessive development."

Dugas* and Dr. Notman Bridget † opposed the theory; and Dr. D. S. Conant, in a paper read before the New York Academy of Medicine, on "Monstrosities," argues that anomalies are due to arrested development of the fœtus, and not to maternal impressions, based on the statement of Virchow that there is no nervous tissue in the umbilical cord, and the only means of communication between the mother and fœtus is through the medium of the blood. In the discussion of the paper, Dr. Detmold opposed the theory, so also the late Dr. Peaslee, both saying that deformity was due to an arrest of development. The latter adds, however, the following: "When the pregnant woman becomes the subject of intense anxiety, fear, or other kinds of mental emotions, the offspring might be more or less impressed. The reason for this effect on the fœtus could be explained by the close sympathy between the brain and uterus. The blood going to an impregnated uterus may be so altered in quality as to produce fatal effects on the fœtus, if, after a pregnant woman is frightened, a monster is produced in a *general* instead of a *special* way by the arrest of development. For it is a well known fact that the fœtus, during its various stages of development, passes through certain conformations which in general appearance very much resemble those of lower animals."

Velpeau, Ryan, Demageon and Reinvielliers entertain similar views.

On the other hand, Montgomery, Rokitansky, Carpenter (physiology), Dalton, Flint, Hammond, Allen Thompson, Fordyce Barker and E. C. Spitzka favor the doctrine of maternal impressions. The latter has written a very able and scientific article, ‡ in which he says:

"Those who discredit the evidence accumulated in support of the doctrine of maternal impressions may be divided into two classes. The first consists of those who,

* Southern Med. and Surg. Journal, Sept., 1886, p. 317.

† Chicago Medical Journal, August. 1875, p. 577.

‡ "Medical Classics" Volume ii, Nos. 2 and 3, Aug. and Oct. 1888.

of a critical and analytical turn of mind, have unearthed so much that is absurd or inconclusive in the related cases, that they have hastily assumed all to be of the same fallacious nature. The second comprises those whom I would designate as 'coarse materialists.' Let this term not be misunderstood! Every scientist is properly a materialist. But there is a class of writers and workers who cannot see beyond their fingers, who, in view of the fact that the great advance of science in this century has been owing to the methods of the laboratory, have gotten up a sort of spurious enthusiasm for the balance, the kymograph, microscope and thermometer, and with an assumption of hauteur, sneer down everything that cannot be weighed, measured, chemically tested or photographed."

Again:

"I have never seen an idiotic, malformed child, or one afflicted wtth morbid impulses, derived from healthy parents of families free from hereditary taint in which a maternal impression could not be traced. In a large number a direct correspondence between the maternal impression and the nature of the deformity or peculiarity could be discovered."

After citing several cases, he holds that the doctrine of maternal impressions is maintained by the following facts:

"1. One class of them is established to be due to such by statistical evidence. 2. Another class is established by the exact reproduction of the maternal impression in the fœtus, so exact that all explanations based on the assumption of a fortuitous occurrence must be rejected. 3. Cases are recorded where the same cause produced the same effect in several instances. 4. Cases are recorded where a multiplicity of impressions on the same mother led to a multiplicity of defects in the offsprtng, all of them reproducing the original impression. 5. As already pointed out, the fœtal defect is in harmony with the embryological law that the earlier the influence is excited in the germ, the deeper its effect—in other words, the greater the deformity. 6. The counter-argument of opponents of the doctrine that the monstrosity is often quite unlike the impression to which it is attributed, is met by the fact just cited, and also by the fact that in ordinary (universally admitted) hereditary transmission the same defect is not necessarily transmitted. The transmission of maternal impressions conflicts in no detail with the laws of embryonic development and heredity. It is in complete harmony with them."

Also:

"One fact speaks strongly in favor of the truth of the doctrine of maternal impressions, and that is their consistency with embryological laws, and even with embryological experimentation. It is noted that in producing monstrosities by artificial methods, an interference with development at an early period produces a far greater malformation than one at a later period. In the foregoing series of cases it may have been remarked by the reader that deep defects, involving grave malformation of the brain, occurred in the first two months of pregnancy. That such involving absence of bony parts occurred before the sixth month, and that all birth-marks attributed to impressions occurring in the last month were at most skin deep. It is sometimes possible by a disturbance of a germ at a very early period of its history, to cause a division, in other words, a conjoined twin monster. All the two-headed children of mothers who had been impressed by the "two-headed nightingale" were attributed to impressions occurring in the first few days after conception, and consistently so, for such a deformity cannot be accounted for by causes operating after the second week."

Dr. Fordyce Barker has written:

It will be observed that all who disbelieve in this doctrine base their skepticism on what they regard as physiological reasoning, and chiefly on the assertion that there is no direct nerve communication between the maternal and fœtal system, and that, therefore, nerve impressions cannot be transmitted to the fœtus. Deformities, they urge, are due to arrest of development, but no one has brought forward any sound physiological reason why this arrest of development may not have been caused by maternal impressions, affecting fœtal nutrition by their influence on the maternal blood, as well as by falls, injuries, diseases, intra-uterine amputations by ligation of the umbilical cord, and the various other causes which have been assigned. Those numerous changes in fœtal development where the effect corresponds with known maternal impressions, of which hundreds have been published, are considered as simply coincidences. But if mathematical calculation could be made as to the chances for such a coincidence, I believe that the oods would be so enormous as to be almost beyond enumeration. My personal acquaintance with the profession leads me to suppose that a very large majority utterly disbelieve in this influence, and I ascribe this skepticism to the fact that, while they find this belief almost universal, to such an extent as to cause great anxiety in many of their patients, especially if they have been subjected to any strong emotion, yet the verification of this apprehension is so extremely rare that probably not one in a hundred ever meets with a convincing case.

"Extremely rare as is the occurrence of cases which prove the result of this influence, yet I think the fact is so well proved by sufficient authentic evidence as to make it as certain as any other fact which cannot be explained by science, and there are many such. Indeed, in the light of all the evidence which has been accumulated on this point, it seems to me as reasonable to deny the occurrence of earthquakes because philosophy has not yet been able to give a satisfactory explanation of their cause."

One of the most frequent deformities impressing the mother's eye and transmitted in exact repetition to the child is hare-lip. Dr. Barker relates a case occurring in his own practice in which the influence of maternal impression seems well grounded.

"A lady was married at the age of twenty, when her father made her a present of a house. She was absent on her wedding trip for two weeks, and then went to the Gramercy Park Hotel to stay while her house was being repainted and decorated, and such furni ure as she wished was selected and purchased. She had not menstruated since her marriage. On her first day at this hotel she went to the table d'hote, and found herself seated opposite a gentleman with three daughters who all had hare-lips. (This family is well-known.) The first glance at them made her so faint that she at once left the table, and always after took her meals in her private rooms until she moved to her own house. She never mentioned her reasons for this even to her husband, nor had she any suspicion that she was then pregnant. I attended her in her confinement, which was a very laborious one, and she was delivered by the forceps, profoundly under the influence of chloroform. I saw at once that the child had a double hare-lip, and sent for Dr. Carnochan, who had finished the operation before she awoke from the chloroform sleep. On becoming conscious she demanded to see her child, saying that she was certain that it had a hare-lip. I refused to allow her to see her child until the next morning, and gave her a full opiate. The operation was remarkably successful, the mother did well, and the child, now nearly thirty, would not attract attention by the appearance of his lip, but only by an indistinct articulation of a few words."

In a foot-note he adds the following:

"I may here say that this is the only case which has occurred in my practice in which any peculiarity of the child had been anticipated by the mother, although the apprehension has been strongly expressed by many."

As to the question of operation upon cases of hare-lip, whether it should be performed immediately or delayed until a later period, he says:

"My decision was in accordance with my action in the present case. The same course was followed in two cases which I saw in consultation, and with a success which caused no regret."

He also relates a case which was told him by his friend, Dr. A. Brayton Ball, of New York.

"Mrs. B , a woman of highly nervous temperament, pregnant between two and three months with her first child, was much startled by seeing a child about ten years old with a hypertrophied, prolapsed tongue. The child's appearance was extremely repulsive, and so shocked Mrs. B., that she nearly fainted. From this time on she was apprehensive that her child would be marked in the same way, and this fear was shared by her aunt, who was present when the incident occurred, though the matter was never afterward referred to between them during the pregnancy. At birth Mrs. B.'s child presented exactly the same deformity. The tongue was hypertrophied, and hung down over the lower lip, but with this exception was perfectly formed. The tongue remained outside of the mouth until the child was several years old, and then gradually retreated into the cavity, but has always remained sufficiently large to interfere with the proper enunciation of words. No similar case has been known in either branch of the family, and several children have been born since then, all perfectly developed. I regret that I cannot state the exact period of pregnancy when the 'maternal impression' was made, as it happened nearly thirty years ago, but the date probably fell between the limits mentioned. Mrs. B., though not a patient of mine at the time, became so afterward, and her account of the case agrees in every particular with that given me by her aunt, who was with her when the incident occurred and at her confinement. I make no comment on the case, except to say that I regard it as in the highest degree improbable that the only relation between the two events is that of mere coincidence."

Dr. Arthur Coe, of Ann Arbor, Mich., reports the following.*

"Mrs. O., aged thirty-two, two children, the elder aged

* Medical Record, October, 20, 1888, page 479.

twelve years, has always enjoyed good health. The left breast presented two nipples, the smaller or supplementary one being about two inches below the normal nipple. It was correctly proportioned and surrounded by a faint areola. The patient stated that during lactation the secretion flowed freely from the lower nipple, requiring the application of a bandage. The possibility of the supposed nipple being a fistulous opening with everted edges resulting from an old abscess, was considered, but a careful examination and a statement by the patient, to the effect that the two nipples had been present as long as she could remember, seem to be conclusive evidence against that possibility. An interesting point in the case centers in the possibility of the malformation being due to a so called 'maternal impression.' Upon inquiry the patient stated that the mother when about two months advanced in pregnancy, was much startled on one occasion by a young kitten, which jumped upon her breast (the left) and seized the skin just below the nipple. She was much prostrated by the occurrence, which made a deep impression on her mind. The subsequent birth of the child with the peculiar malformation would seem to indicate some dependence thereon."

The cases that could be brought forward in which the theory of maternal impressions seems irresistible are numerous. It is difficult to account for so singular a transmission, as we know of no direct channel of nervous communication, nor can we assume that the circulating fluids can carry or interchange such influence. But let us not voluntarily blind ourselves to facts, as a "future with vastly larger knowledge of physiology and psychology may plainly read the mysteries hidden from our sight."—*Albany Medical Annals*.

